# Longitudinal transcriptome analyses show robust T cell immunity during recovery from COVID-19

**DOI:** 10.1038/s41392-020-00457-4

**Published:** 2020-12-24

**Authors:** Hong-Yi Zheng, Min Xu, Cui-Xian Yang, Ren-Rong Tian, Mi Zhang, Jian-Jian Li, Xi-Cheng Wang, Zhao-Li Ding, Gui-Mei Li, Xiao-Lu Li, Yu-Qi He, Xing-Qi Dong, Yong-Gang Yao, Yong-Tang Zheng

**Affiliations:** 1 Key Laboratory of Animal Models and Human Disease Mechanisms of the Chinese Academy of Sciences and Yunnan Province, KIZ-CUHK Joint Laboratory of Bioresources and Molecular Research in Common Diseases, Center for Biosafety Mega-Science, Kunming Institute of Zoology, Chinese Academy of Sciences, Kunming, Yunnan 650223 China; 2 Yunnan Infectious Disease Hospital, Kunming, 650301 China; 3 Kunming Biological Diversity Regional Center of Large Apparatus and Equipments, Public Technical Service Center, Chinese Academy of Sciences, Kunming, Yunnan 650223 China; 4 Kunming College of Life Science, University of Chinese Academy of Sciences, Kunming, Yunnan 650204 China; 5 CAS Center for Excellence in Brain Science and Intelligence Technology, Chinese Academy of Sciences, Shanghai, 200031 China

**Keywords:** Infectious diseases, Genome informatics

## Abstract

Understanding the processes of immune regulation in patients infected with the severe acute respiratory syndrome coronavirus 2 (SARS-CoV-2) is crucial for improving treatment. Here, we performed longitudinal whole-transcriptome RNA sequencing on peripheral blood mononuclear cell (PBMC) samples from 18 patients with coronavirus disease 2019 (COVID-19) during their treatment, convalescence, and rehabilitation. After analyzing the regulatory networks of differentially expressed messenger RNAs (mRNAs), microRNAs (miRNAs) and long non-coding RNAs (lncRNAs) between the different clinical stages, we found that humoral immunity and type I interferon response were significantly downregulated, while robust T-cell activation and differentiation at the whole transcriptome level constituted the main events that occurred during recovery from COVID-19. The formation of this T cell immune response might be driven by the activation of activating protein-1 (AP-1) related signaling pathway and was weakly affected by other clinical features. These findings uncovered the dynamic pattern of immune responses and indicated the key role of T cell immunity in the creation of immune protection against this disease.

## Introduction

Since December 2019, a new zoonotic severe acute respiratory syndrome coronavirus 2 (SARS-CoV-2) has swept the world, causing a variety of clinical syndromes collectively termed coronavirus disease 2019 (COVID-19).^1–3^ The World Health Organization declared a pandemic in March 2020. The symptoms of COVID-19 are fever, dry cough, fatigue, diarrhea, conjunctivitis, and pneumonia.^1^ Most people do seem to be less affected, either remaining totally asymptomatic or having only mild symptoms. However, some people develop a severe pneumonia, acute respiratory distress syndrome (ARDS) or multiple organ failure.^2,4^ It is currently believed that severe COVID-19 pathogenesis may be mediated by a unique immune response disorder, and the host antiviral immune response affects the severity of the disease and the clinical outcome.^5,6^

The immune pathology caused by SARS-CoV-2 and the immune protection against COVID-19 had received extensive attention. Recent studies had shown that immune system disorders, such as lymphocytopenia and inflammatory cytokine storm, are associated with the severity of the SARS-CoV-2 infection.^1,7^ Type I interferon (IFN-I) not only has the ability to clear the virus, but also can cooperate with inflammatory factors to promote the severe development of COVID-19.^8,9^ In particular, the inflammatory, exhausted and activated state of T cells affects the severity of COVID-19 symptoms, whereas a robust T cell immune response in patients may affect the predisposition to disease and also prevents re-infection.^10–12^ The humoral immunity against SARS-CoV-2 works rapidly but may not provide long-lasting immunity, as revealed by recent evaluation of the decay of anti-SARS-CoV-2 antibodies in patients with COVID-19.^13,14^ These studies had greatly expanded our understanding of COVID-19 pathophysiology and immunology. However, we are still unclear about the main events of the immune regulation process of patients during their recovery from illness.

Transcriptome analyses are very suitable for the study of viral infection immunology and allow for an understanding of the immune response dynamics and gene regulatory networks. Recent transcriptome analyses of patients with COVID-19 have shown the dynamics of the immune responses following infection with SARS-CoV-2.^6,7,9,15,16^ Here we characterized the longitudinal transcriptome changes in peripheral blood mononuclear cells (PBMC) of 18 COVID-19 patients with mild, moderate or severe symptoms at three clinical stages (treatment, convalescence, and rehabilitation). Our results showed the immune remodeling processes in patients at the different stages of their illness and revealed the core role of T cell immunity.

## Results

### Clinical features of SARS-CoV-2 infected patients with mild, moderate or severe symptoms during recovery from COVID-19

**Fig. 1:**
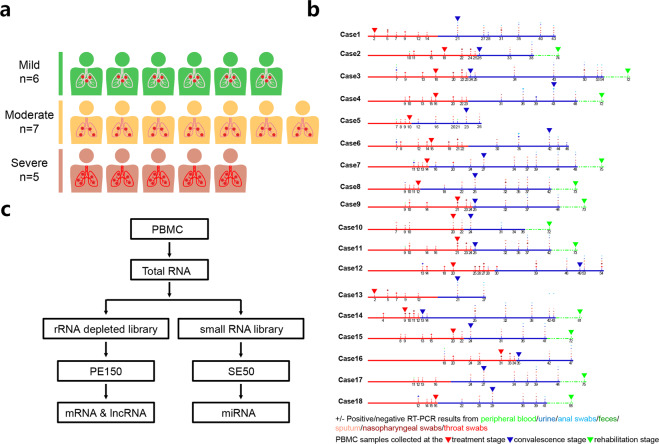
Study design of whole transcriptome sequencing for PBMCs from patients with COVID-19 during recovery. **a** Patients are divided into mild, moderate and severe groups according to clinical symptoms of each one. **b** Timeline of the disease course of 18 patients infected with SARS-CoV-2. RT-PCR indicates the PCR test of SARS-CoV-2 nucleic acid. A positive RT-PCR (marked by “+”) indicates that the SARS-CoV-2 nucleic acid was found in the throat swab, nasopharyngeal swab, sputum or other samples, which were distinguished by using different colors. “−”, negative RT-PCR. The solid red lines stand for treatment stage, solid blue lines for convalescence stage and doted green lines for rehabilitation stage. The sampling days were marked below this line. The inverted triangle symbol is used to mark the time point for collecting PBMC samples at each stage. **c** A flowchart showing the process of library construction and RNA sequencing of PBMC samples from patients with COVID-19

From January 17 to February 19, 2020, 18 patients (11 male and 7 female) at the Yunnan Infectious Disease Hospital, Kunming, China, with COVID-19 and SARS-CoV-2 infection confirmed by laboratory testing, were recruited in this study. According to the guidelines for diagnosis and management of COVID-19 (6th edition) issued by the National Health Commission of China, 5 patients (severe group, *n* = 5) developed severe pneumonia by imaging examination, with the percutaneous oxygen saturation (SpO_2_) less than 93% or respiratory rate (RR) exceeding 30 breaths/min in the resting state, and were diagnosed as severe COVID-19; 7 patients (moderate group, *n* = 7) had fever, dyspnea, and other respiratory symptoms, with computed tomography (CT) imaging findings of pneumonia, but did not meet the severe criteria, and were classified as having moderate COVID-19; the remaining 6 patients (mild group, n = 6) were diagnosed as mild based on their milder clinical symptoms and no obvious pneumonia (Fig. 1a).

Among these patients, 16 were treated with the recombinant human IFN-α-2b with/without other antiviral drugs immediately after admission, and 2 patients in the mild group were treated with arbidol hydrochloride instead of IFN-α-2b. After the body temperature was normal for more than 3 days and at least two consecutive SARS-CoV-2 nucleic acid tests for respiratory samples (throat swab, nasopharyngeal swab, and sputum) were negative at least one day apart, the patients were isolated and entered the convalescence stage. From February 24 to April 2, 2020, all the patients were discharged from the hospital in batches and entered the rehabilitation stage based on the following criteria: the respiratory symptoms had improved significantly, SpO_2_ and RR returned to normal in severe patients, CT imaging of the lungs showed obvious absorption of inflammation, and the nucleic acid tests were negative on 2 consecutive occasions (Fig. 1b, Supplementary Table 1). Blood samples were collected at appropriate times for our later studies.

### Enhanced immune regulation drives the immune system remodeling during recovery from COVID-19

**Fig. 2:**
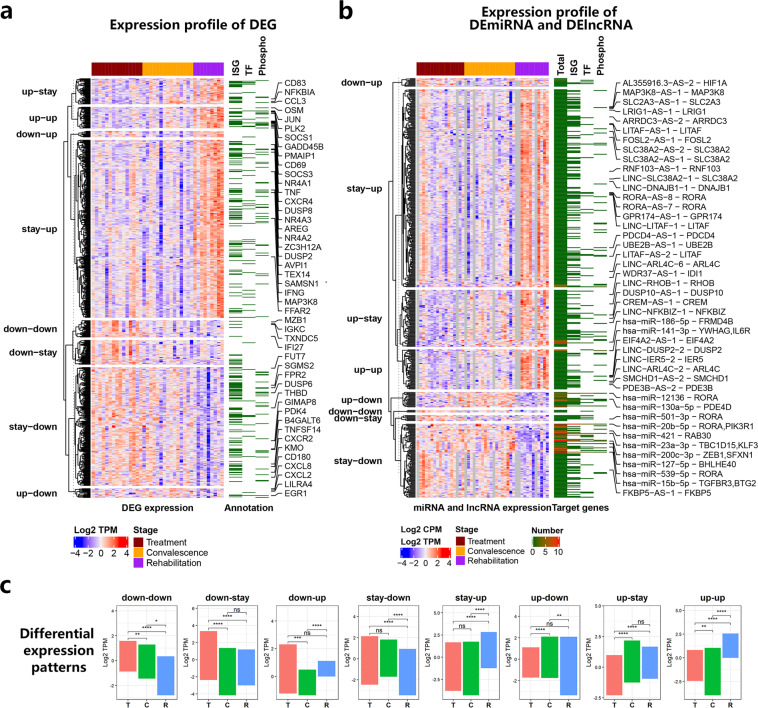
Differentially expressed genes (DEG), miRNAs (DEmiRNA), and lncRNAs (DElncRNA) in PBMC samples from patients with COVID-19 during the recovery. **a** A heatmap showing the expression profiles of 643 DEGs. The blue-red gradient square maps the scaled log_2_ value of transcripts per kilobase million (Log_2_ TPM) for each DEG in a sample. The grouping information on the left indicates genes with different differential expression patterns. The colored bars above the heatmap refer to the clinical stage of each sample. The gene function annotation on the right marks the interferon-stimulated genes (ISG), transcription factor (TF) and phosphorylation regulatory genes (Phospho) in DEGs. The hub genes with a more significant differential expression in the function networks constructed by all DEGs are marked with the respective gene names. **b** A heatmap showing the expression profiles of 67 DEmiRNAs and 405 DElncRNAs. The blue-red gradient square maps the scaled log_2_ TPM of each DElncRNA, or the scaled log_2_ value of counts per million (Log_2_ CPM) of each DEmiRNA in a sample. The gene function annotation shows the number of target genes for each non-coding RNA. When the target gene is a DEG, we annotate the regulation module in the format of “non-coding RNA name - target gene name” on the right. **c** By comparing the *P* value and TPM/CPM at three different clinical stages, DEG, DEmiRNA or DElncRNA can be further clustered into 8 expression patterns. Each pattern reflects the characteristics of its RNAs being upregulated (up), downregulated (down) or stayed for no change (stay) at the convalescence (C) and rehabilitation (R) stages relative to the treatment (T) stage. Ns, *, **, ***, ****: *p* > 0.05, ≤0.05, ≤0.001, ≤0.0001, respectively

We obtained the whole transcriptome data of 47 PBMC samples from 18 patients with COVID-19 during the treatment, convalescence, and rehabilitation stages (Fig. 1c). Clinical stage-related 643 differentially expressed genes (DEGs; by 39 samples), 405 differentially expressed long non-coding RNAs (DElncRNAs; by 39 samples) and 67 differentially expressed microRNAs (DEmiRNAs; by 47 samples) were identified based on the rule of false discovery rate (FDR) < 0.05 (Fig. 2a, b; Supplementary Fig. 1). The linear regression slope of the log2 fold change of DEG, DEmiRNA target genes (*n* = 309) and DElncRNA regulated genes (*n* = 405) was further performed on Gene Set Enrichment Analysis (GSEA).^17^

**Fig. 3:**
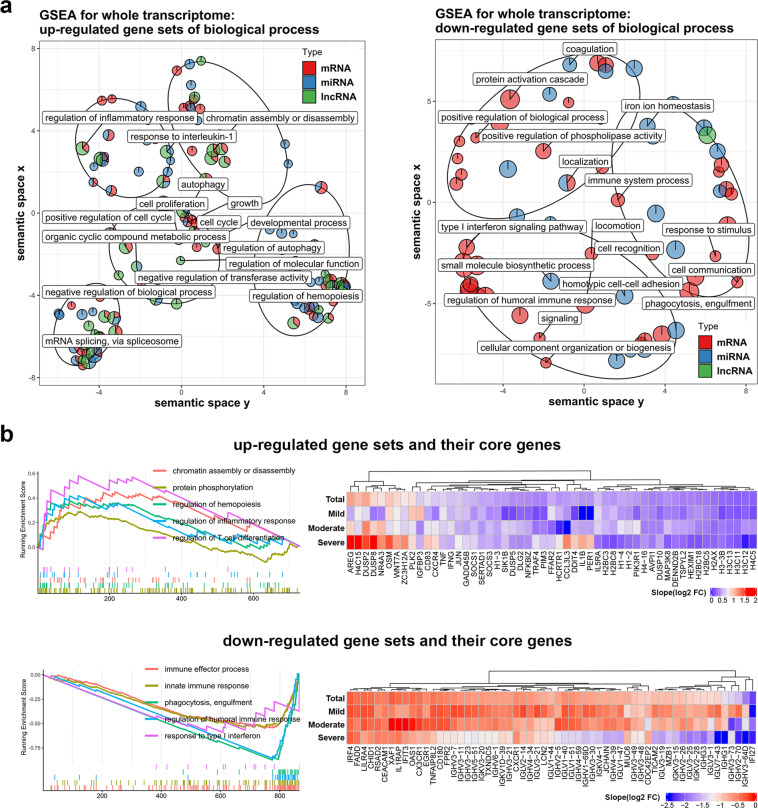
Gene set enrichment analysis (GSEA) showing an immune system remolding at the whole transcriptome level. **a** The bubble-pie graphs displaying upregulated and downregulated gene ontology (GO) gene sets enriched by the differentially expressed mRNAs, miRNAs, and lncRNAs. The color refers to the indicated type of transcripts enriched for a gene set. The bubble size maps the sum enrichment scores of all RNA types in a gene set. The X and Y values of a gene set are calculated by REVIGO’s GO semantic space algorithm.^69^ The ellipses refer to clusters formed by gene sets with a high semantic similarity. **b** GSEA plot showing the distribution of the upregulated or downregulated gene sets and the enrichment scores based on DEGs for all samples. The heat map shows the core genes in these gene sets of different clinical stages for all samples, mild, moderate and severe groups, respectively. The blue-red gradient square maps the slope of the linear regression equation of Log2 fold change of DEG

The gene ontology (GO) semantic clustering analysis showed that the gene sets enriched by differentially expressed coding and non-coding RNAs did not differ in main categories. Only the gene sets of regulation of hemopoiesis, regulation of inflammatory response, mRNA splicing via spliceosome and epithelial cell proliferation were upregulated at the whole transcriptome level during recovery from COVID-19 (Fig. 3a; Supplementary Fig. 2), which contained many transcription factor genes (e.g., *DDIT3, NR4A3, ZEB1, KLF10, JUNB*, and *JUN*), phosphorylation regulatory genes (e.g., *AREG, DUSP8, OSM, MAP3K8*, and *SOCS3*), and interferon-stimulated genes (ISG; e.g., *TNF, IL1B, CXCR4, CD69*, and *IER5*) (Fig. 3b). The innate immune response represented by the type I interferon (IFN-I) signaling pathway and the humoral immune response represented by B cell receptor signaling pathway were grouped into downregulated gene sets that are enriched with DEGs and DEmiRNAs (Fig. 3a; Supplementary Fig. 2). A large number of immunoglobulin genes were downregulated, in addition to the downregulation of antiviral immune genes such as *IFI27, OAS1, IFIT3, FADD*, and *RSAD2* (Fig. 3b). These observations suggested that immune regulation plays a key role in the remodeling of innate and adaptive immunity during the recovery from COVID-19.

A large number of gene sets involved in immune cell differentiation were also found to be upregulated during recovery from COVID-19, including autophagy, cell cycle, cytokine production, regulation of T cell differentiation, response to interleukin-1, immune system development, and regulation of NIK/NF-κB signaling. Furthermore, decrease of the enrichment scores of the gene sets related to blood coagulation and platelet activation also reflected that these comorbidities of abnormal blood coagulation are being reduced in these patients (Fig. 3a; Supplementary Fig. 2).

### Elevated T cell activation and differentiation are core events during recovery from COVID-19

**Fig. 4:**
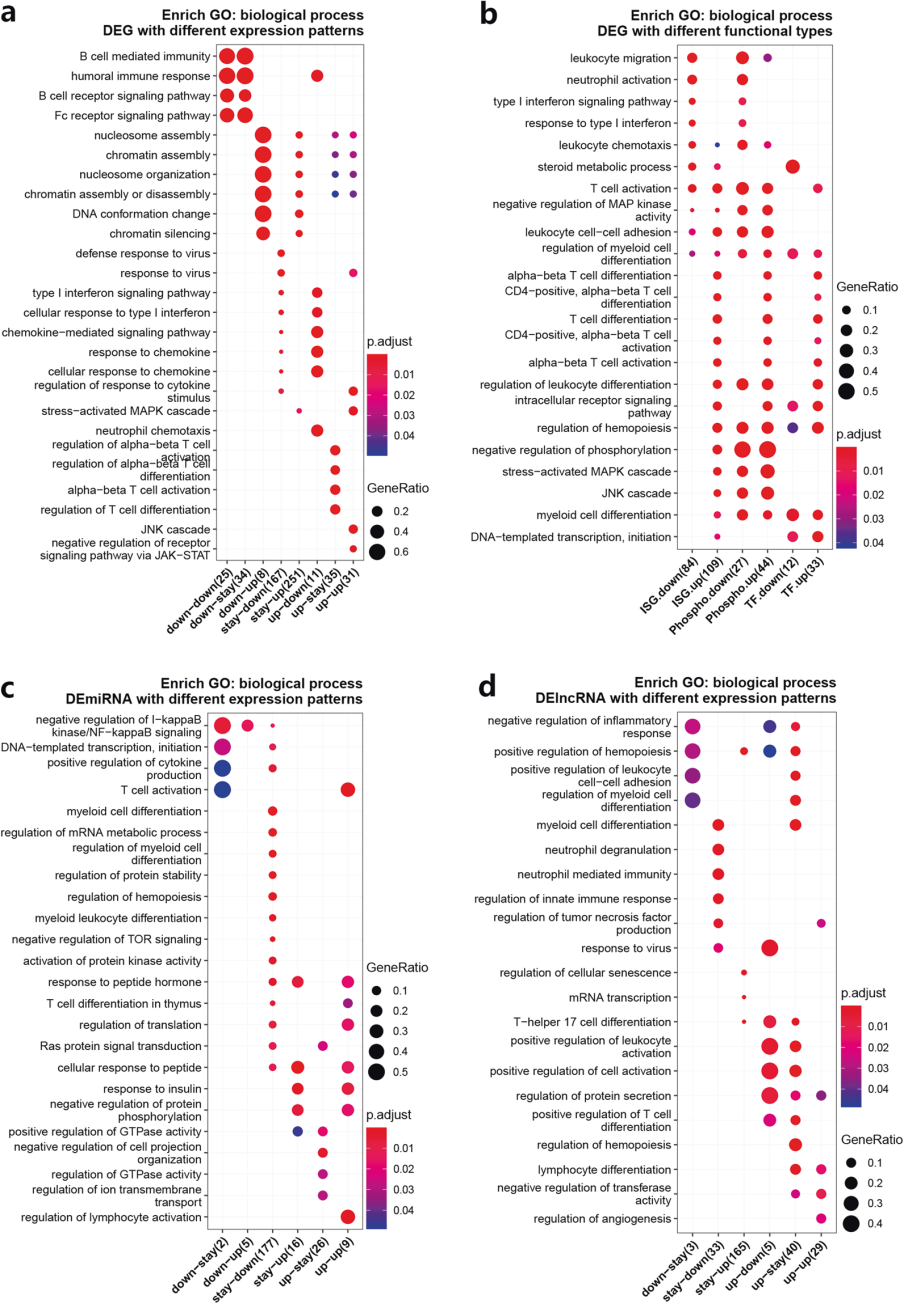
Gene ontology (GO) enrichment analysis identified T cell immunity as the main regulatory target during the recovery of COVID-19. **a** GO enrichment results of DEGs in 8 differential expression clusters defined in Fig. 2b. The numbers of significantly enriched genes in each cluster were included in parentheses. **b** GO enrichment results of interferon-stimulated genes (ISG), transcription factor genes (TF), and phosphorylation regulatory genes (Phospho) in DEGs. **c**, **d** GO enrichment results of the genes targeted by DEmiRNAs (**c**) and DElncRNAs (**d**) in 6 differential expression clusters defined in Fig. 2c. The bubble size indicates the gene enrichment ratio (Generatio) of a biological process GO term, with color maps the FDR value (p.adjust) of the enrichment analysis

A total of 8 differential expression patterns were clearly discerned in patients with COVID-19 at the treatment, convalescence, and rehabilitation stages, respectively (Fig. 2c). Using enrichment analysis grouped by these expression patterns, we found that DEGs, which are downregulated only at the rehabilitation stage (stay-down), enriched by a large number of antiviral genes and IFN-I signaling genes (e.g., *OAS1, IFIT3, AIM2, IFIT1*, and *FADD*), positive regulatory genes of cytokine signaling pathway (e.g., *TLR2, IL6R, P2RX7, RSAD2* and *AIM2*), as well as some chemokine receptor genes (e.g., *CCR1, CCR5, CMKLR1, CX3CR1*, and *CXCR2*). However, humoral immunity was attenuated earlier than innate immunity. DEGs that downregulated from the convalescence stage to the rehabilitation stage (down-stay or down-down) were enriched in GO terms such as humoral immunity, B cell immunity, B cell activation and Fc receptor signaling (Fig. 4a, Supplementary Fig. 3).

One prominent feature of the enrichment analysis was that many DEGs upregulated in the convalescence, and rehabilitation stages (stay-up or up-stay) are involved in T cell activation and differentiation, such as *PIK3R1, IFNG, CYLD, RHOH, BCL3, MAP3K8*, and *ICOS*. Moreover, a group of DEGs that upregulated in the convalescence stage and then continued to be upregulated in the rehabilitation stage (up-up) were mainly composed of genes that regulate protein kinase activity, including *TNF, CXCR4, SOCS1, JUN, SOCS3, PDCD4, DUSP8*, etc. These genes are actively involved in the regulation of MAP kinase activity and JAK-STAT signaling pathway (Fig. 4a, Supplementary Fig. 3), suggesting that the T-cell immunity was elevated during recovery from COVID-19 under a chronological regulation. Only 69 DEGs showed significant differences between each comparison of mild, moderate and severe groups. These genes were mainly enriched in GO terms of negative regulation of phosphorylation, leukocyte migration and regulation of hematopoiesis (Supplementary Fig. 4).

### Multiple levels of regulation participate T cell activation and differentiation during recovery from COVID-19

Further enrichment analysis of DEGs related to interferon, transcription factor and phosphorylation regulation revealed that the activation and differentiation of T cells are the main targets of their regulation. *PRDM1* (up-stay), *RORA* (stay-up), *BCL6* (up-stay), and *ZEB1* (stay-up) acted as important transcription factors for controlling the differentiation of T cells and mediating the differentiation of tissue-resident T cells, Th17 cells, follicular helper T cells, and memory T cells, respectively.^18–21^ For phosphorylation regulatory DEGs, *IFNG* (stay-up), *DUSP10* (up-stay) and *SOCS1* (up-up) were enriched in regulatory T cell differentiation, while stay-down genes *FCGR2B*, *CEACAM1*, and *P2RX7* were enriched in T cell mediated cytotoxicity. Upregulated ISGs, such as *RIPK2* (up-stay), *IFNG* (stay-up), *CD83* (up-stay) and *SOCS1* (up-up), were mostly enriched in T cell activation. Among them, *RORA* (stay-up), *NFKBIZ* (up-stay) and *ZC3H12A* (stay-up) were enriched in Th17 differentiation, while *BCL3* (stay-up) and *BCL6* (up-stay) were enriched in Th2 differentiation (Fig. 4b).

Consistent with the changes of DEGs, there were many upregulated DElncRNAs that positively regulate the differentiation of lymphocytes and T cells at the convalescence and rehabilitation stages. The regulation of T cell differentiation by DEmiRNAs mainly occurred at the rehabilitation stage. The stay-down miRNAs let-7b-5p, miR-103a-2-5p, miR-200c-3p and miR-2115-3p were significantly downregulated, while their target genes (*RASGRP1*, *CDK6*, *ZEB1*, and *ATG5*, respectively) were significantly upregulated. The stay-up DElncRNAs RORA-AS-7, RORA-AS-8, ITPKB-AS-1, RUNX1-AS-8, STAT3-AS-1, and MALT1-AS-2 were enriched in differentiation of T-helper cells. In addition, the stay-up DElncRNAs MAP3K8-AS-1, RASGRP1-AS-1, RASGRP1-AS-2, LINC-CD47-1, LINC-CD44-1, PREX1-AS-2, TNFRSF1B-AS-1, and TNFRSF1B-AS-2 were enriched in T cell activation (Fig. 4c, d).

### The AP-1 linked signaling pathway plays a key role in T cell activation and differentiation during recovery from COVID-19

**Fig. 5:**
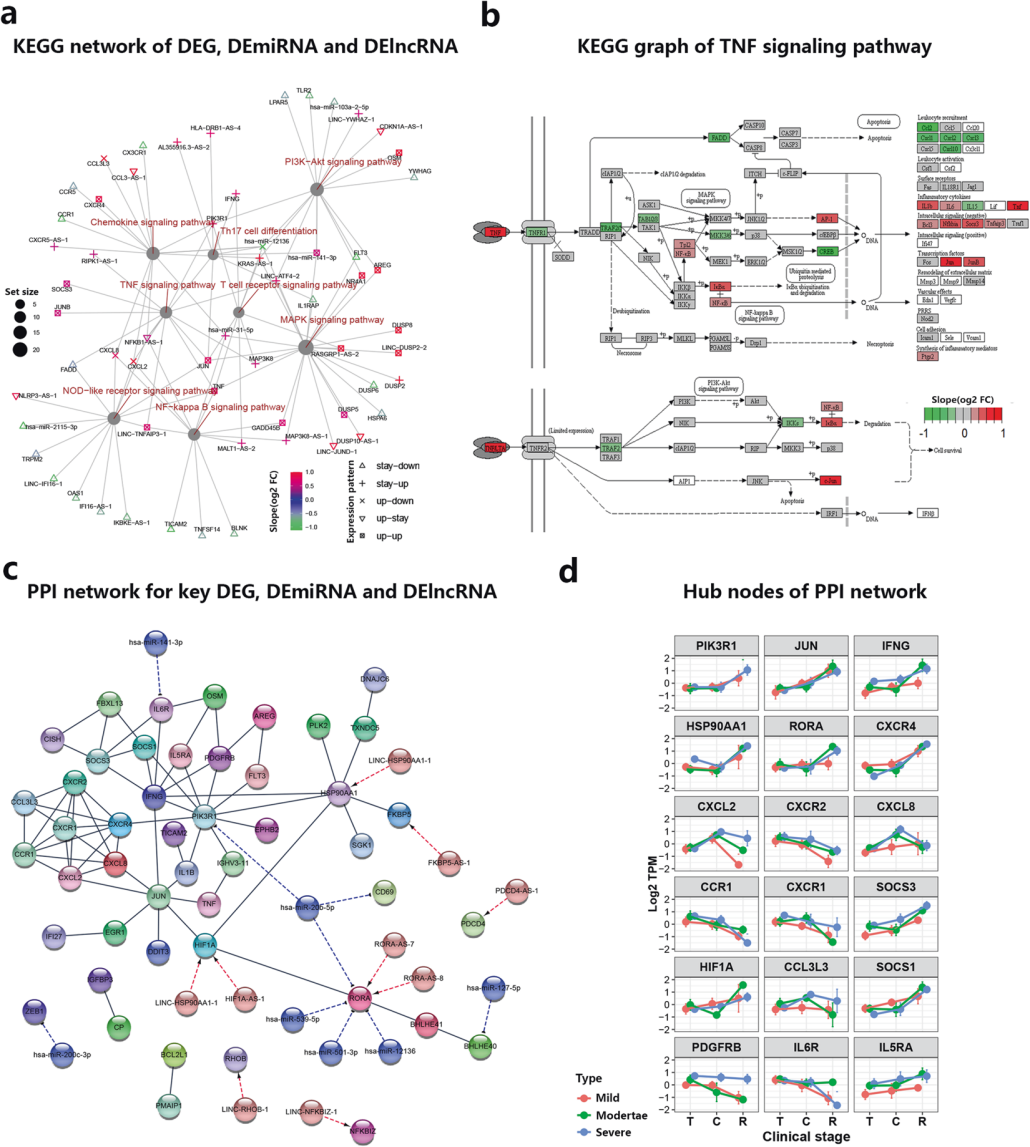
Gene function network revealed a core role of the AP-1 linked signaling pathways during the recovery of COVID-19. **a** A network showing the relationship between genes and enriched KEGG pathway. The genes displayed are selected from the DEGs and the genes regulated by DEmiRNAs or DElncRNAs with a more significant differential expression and a higher connectivity. The color maps the linear regression slope of Log_2_ fold change of a gene. The size of the circle is proportional to the number of genes enriched in a KEGG pathway. **b** A KEGG graph showing the interactions of genes involved in the TNF signaling pathway. The color indicates linear regression slope of Log_2_ fold change of a gene. **c** A protein–protein interaction network (PPI) showing the interactions between genes and non-coding RNA. The genes displayed are selected from the top hub genes with the most significant differential expression. **d** A line graph showing the expression profiles of the main hub genes during COVID-19 recovery. The color indicates samples with different clinical types. T, treatment stage; C, convalescence stage; R, rehabilitation stage. The data are shown as mean ± SD

Using the functional network analyses of DEGs, DEmiRNAs and DElncRNAs, we found that the TNF, MAPK, and NF-κB signaling pathways are the most significantly changed pathways during recovery from COVID-19 (Supplementary Fig. 3-6). These networks are closely linked with inflammatory factor genes *TNF* and *IL1B*, and transcription factor AP-1 subunit gene *JUN*. The AP-1 connected nodes were at the core of the enriched KEGG maps, including Toll-like receptor signaling pathway, IL-17 signaling pathway and PD-L1 expression and PD-1 checkpoint pathway (Fig. 5a). The activation of AP-1 and NF-κB signals caused an increased expression of inflammatory factors such as TNF-α, IL-1β, IL-8, MIP-1α and MIP-1β according to the KEGG enrichment analysis (Supplementary Fig. 7), which might be detrimental to COVID-19 rehabilitation. Concordantly, for downstream genes of AP-1, the expression levels of some chemokine genes (e.g., *CCL2, CXCL1, CXCL2*, and *CXCL10*) were downregulated, while the expression levels of immune negative regulation genes (e.g. *BCL3, NFKBIA, SOCS3*, and *TNFAIP3*) were upregulated during the COVID-19 recovery (Fig. 5b). These results indicated that the activation of the AP-1 linked pathway does not cause excessive inflammation and was more likely to play a role in immune regulation.

The transcription factor AP-1 subunit genes *FOSL2* and *JUNB* were also upregulated at the rehabilitation stage, and the non-coding RNAs (e.g., FOSL2-AS-1, LINC-JUND-1, and miR-494-3p) that regulate these genes were also significantly differentially expressed. Some genes that interact with *JUN*, such as *HIF1A*, can enhance the effector function of T cells and promote virus clearance,^22^ and were regulated by DElncRNAs HIF1A-AS-1 and LINC-HSP90AA1-1. Other genes need to work with AP-1 for controlling T cell differentiation, such as *RORA*,^23^ were regulated by a variety of DElncRNAs, including RORA-AS-7, RORA-AS-8, miR-20b-5p, miR-12136, miR-539-5p, and miR-501-3p (Fig. 5c). In addition, we found that *JUN, PIK3R1, IFNG, CXCR4, SOCS3, HIF1A*, and other significantly regulated genes had the highest connectivity in the GO, KEGG, and protein–protein interaction (PPI) networks (Fig. 5d). Therefore, the AP-1 linked signaling pathway acted as a core to regulate T cell activation and differentiation at whole transcriptome level during recovery from COVID-19.

### The activation and differentiation of T cells are associated with various clinical features in patients with COVID-19

**Fig. 6:**
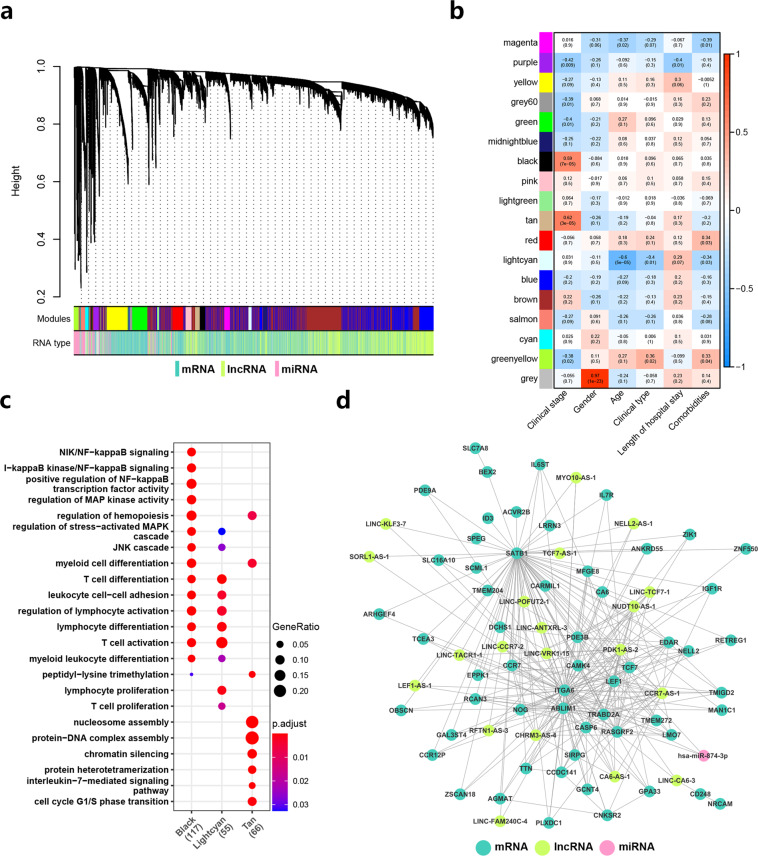
Weighted correlation network analysis (WGCNA) showed the impact of multiple clinical features on the transcriptomic profiling of peripheral blood cells from patients with COVID-19 during the recovery. **a** A hierarchical clustering tree showing all modules based on top 8000 most variably expressed mRNAs/lncRNAs and top 600 most variably expressed miRNAs in all samples. **b** A matrix plot showing the correlations between modules (*Y* axis) and clinical factors (*X* axis). The color and the number outside the brackets of a square indicate the Pearson’s correlation *r* value between a module and the corresponding clinical factor, and the number inside the brackets indicates the Pearson’s correlation *p* value. **c** A bubble plot showing the GO enrichment analysis results of the black, light cyan and tan modules. The numbers in brackets on the *X* axis indicate the total number of significantly enriched genes in each module. The bubble size indicates the gene enrichment ratio (GeneRatio) of a GO term, with color referring to the FDR value (p.adjust) of enrichment analysis. **d** A correlation network showing the co-expression pattern between genes and non-coding RNAs in the light cyan module

To explore the impact of other clinical features on recovery-related whole-transcriptome, we made an analysis of the whole transcriptome using a weighted correlation network analysis (WGCNA). A total of 18 expression modules were discerned, in which the black and tan modules were significantly positively correlated with the clinical stage, but had no significant correlations with other clinical features. Oppositely, significant negative correlations with age, clinical type, and comorbidities were found in the light cyan module, but it did not show a significant correlation with the clinical stage (Fig. 6a, b). It seemed that the transcriptome changes during the recovery process are weakly affected by other clinical features. Specifically, the black module contained many genes and non-coding RNAs related to the regulation of phosphorylation, hematopoiesis and transferase activity, such as *ZC3H12A, DUSP8, CD83, JUNB, NR4A3, OSM, SOCS3, JUN, TNF*, MAP3K8-AS-1, RIPK1- AS-1, and FOSL2-AS-1. The transcripts grouped into the tan module were mostly involved in nucleosome assembly and cell cycle, such as *PLK2, ID2, H4C15, H3C1, H1-2, H1-3*, LINC-H2BC12-1, RCC1-AS-1, and ID2-AS-1. Many genes in these two modules were differentially expressed genes as described above. Although the GO terms enriched by RNAs in the light cyan module overlapped with the black module, it was more concentrated on GO terms related to T cell activation, T cell differentiation, lymphocyte proliferation and myeloid leukocyte differentiation (Fig. 6c). We also discerned the co-expression pattern of genes belonging to the light cyan module. The hub genes of the light cyan module were enriched in T cell differentiation, such as *SATB1, LEF1, CCR7, CAMK4, IL6ST, TCF7, IL7R, SIRPG*, and *TMIGD2*, and lncRNAs of CCR7-AS-1, LEF1-AS-1, LINC-CCR7-2, LINC-TCF7-1 and TCF7-AS -1, and most of them were mainly expressed in low-level differentiated T cells (Fig. 6d).^24–27^ The results showed that various clinical features affect the patient’s T cell immunity, but cannot change its tendency to become robust during recovery from COVID-19.

## Discussion

In this study, we systematically analyzed the whole transcriptome characteristics of PBMC samples from COVID-19 patients with mild, moderate, and severe symptoms at three different time points during their treatment, convalescence, and rehabilitation. The results showed a robust T-cell immune response, but a weakening innate and humoral immunity during recovery from illness, regardless of the clinical types or disease severity, as demonstrated by the altered levels of mRNAs, miRNAs and lncRNAs involved in T cell activation and differentiation.

Many viruses, including SARS-CoV-2, have evolved escape mechanisms against the antiviral activity of IFN-I.^28^ Recent studies had shown that although the production of TNF and IL-6 was increased, and the NF-κB-driving inflammatory response was highly activated, the IFN-I response in seriously ill COVID-19 patients and rhesus monkeys infected with SARS-CoV-2 were still impaired.^9,29^ These findings indicated that IFN-I should be treated as a target for the treatment of COVID-19. In fact, the patients in our study received IFN-α treatment immediately on their admission to hospital and achieved good results. The IFN-I treatment could enhance the cytotoxic function of the patient’s NK and CD8+ T cells,^30,31^ inhibited hematopoietic function and caused the alteration of peripheral blood neutrophils and thrombocytopenia.^32^ In addition, IFN-I treatment directly or indirectly triggered the signal cascade of JAK-STAT, MAPK, PI3K, and NF-κB pathways to participate in the regulation of interferon response.^33–35^ Therefore, the changes at the transcriptome levels of our patients might partially come from interferon therapy. However, despite the fact that the patient’s MAPK and NF-κB signaling pathways were still active, a lot of ISG and the gene sets of positive regulation of T cell cytotoxicity and JAK-STAT signaling pathway were downregulated at the rehabilitation stage, suggesting that the robust regulation of immune response may not directly come from interferon therapy.

Humoral immunity plays an important role in the prevention of infectious diseases, but its role in COVID-19 is still unclear. A detailed clinical report showed that the circulating antibody-secreting B cells appeared in the blood on the 7th day, and IgM and IgG antibodies gradually increased from the 7th day after infection and maintained high levels until the 20th day.^36^ However, multiple studies found that in the first 3 months after infection, the level of antibodies against SARS-CoV-2 in patients who have recovered from mild symptoms reduced sharply, with a predicted exhaustion within a year.^13,37^ These findings mean that although antibodies against SARS-CoV-2 appear quickly, most people may not have lasting immunity. The most recent report on re-infection of SARS-CoV-2 in a human seemed to support this speculation.^38^ In our study, we found that the humoral immune response was attenuated at the convalescence stage (~20–40 days), and there are no significant differences between patients with varying clinical features. The fast decline of humoral immunity against COVID-19 will pose challenges to vaccination, and the detailed mechanism underlying this awaits further study.

A large number of studies have shown that SARS-CoV-2 infection can cause a more pronounced lymphocytopenia in patients with moderate and severe COVID-19, as reflected by the decreased level of T cells, including Th1, Treg, and CD8^+^ T cells.^1,39,40^ In addition, neutrophils increased and monocytes decreased in COVID-19 patients.^39,41^ These abnormalities may be reversed in some immune cells after recovery. For example, Sekine, et al.^42^ found that the SARS-CoV-2 specific T cells in acute infection showed a highly activated cytotoxic phenotype, while these T cells in the recovery stage had multiple functions and exhibited a stem cell-like memory phenotype; Wen, et al.^43^ showed that the T and B cell clones were highly expanded in COVID-19 recovery. In our study, we also found an enhanced transcription of genes involved in the activation and differentiation of T cells during the recovery from COVID-19, indicating that T cell immunity was strengthened.

Further analysis of the functional network of differentially expressed whole transcriptomes showed that AP-1 signal connects MAPK signal and T cell function and plays a key regulatory role. The MAPK signal cascade is essential for regulating AP-1 transcriptional activation and DNA binding activity, while AP-1 has a pleiotropic effect on the process of T cell activation, differentiation, and exhaustion.^44^ The synergy of NFAT and AP-1 would stimulate gene expression after immune response, including IL-2, IFN-γ, TNF-α, GM-CSF, IL-4, FasL, CD25.^45^ The lack of AP-1 led to the repression of downstream genes and blocked the activation and proliferation of T cells, eventually leading to T cell anergy.^46^ Note that the gene set of chromatin assembly was found to be significantly upregulated during the recovery process. Since AP-1 is present in most activated specific open chromatin regions, the binding of AP-1 and the opening of chromatin during T cell activation are conducive to the formation of super enhancers, and the activity of AP-1 helps the activated T cells to form a specific epigenome.^47^ In our study, we found that active T cell immunity is the main immune feature during recovery from COVID-19, which is compatible with a strong AP-1 signaling.

The impact of the clinical features of COVID-19 patients on their immune response has always received much attention. Several recent studies found that the transcriptome of lung tissue and peripheral blood from severe COVID-19 patients was significantly enriched in a large number of ISGs for IFN-I response and a high level of IFN-α, TNF-αand IL-6 compared to healthy people and mild patients.^8,48^ Regarding immune cell function, the loss of the polyfunctionality and the increased level of exhaustion of T cells were important characteristics that distinguish severe from mild patients.^49^ The expression of genes induced by SARS-CoV-2 infection increased with aging.^50^ At the same time, the activation of the innate immune system, the disorder of the adaptive immune system and the inflammatory signal of the elderly were also increasing.^51^ These reasons might account for a higher susceptibility to SARS-CoV-2 in the elderly. There was evidence that during SARS-CoV-2 infection, female patients have stronger levels of T cell activation than male patients, while male patients have higher plasma levels of natural immune cytokines (such as IL-8 and IL-18).^52^ The mechanism by which chronic metabolic comorbidities (such as obesity, type 2 diabetes, and metabolic syndrome) affect COVID-19 has not been fully elucidated, but it was generally believed that it interacts with age and gender to cause immune metabolic disorders and chronic systemic inflammation, which aggravate the excessive inflammation induced by SARS-CoV-2 infection.^53^ In our study, there were very few DEGs between groups of different clinical types during recovery from COVID-19. The results of WGCNA showed that other clinical features have limited influence on the trend of clinical stage-related transcriptome changes. The pattern that patients would have the same strong T cell immune response after recovery from COVID-19 is very promising for the prevention and treatment of COVID-19 and is conducive to the maintenance of herd immunity.

The current study has several limitations. First, we did not perform a fine-grained immune infiltration analysis of immune cell subpopulations using flow cytometry or single-cell analysis in this study, simply because we did not have sufficient samples for these assays. In addition, the altered expression patterns of the hub genes were not validated by quantitative real-time PCR. Second, as the AP1 signaling is at the core and regulates T cell phenotype, the comparison of T cell phenotypes at different clinical stages, and correlation analysis between AP1 signal and infiltration abundance or markers of different T cell phenotypes would further enhance the pattern in this study. Third, we had no complete data of the immunological analyses, such as cytokines, antibodies, and biochemical indices, for these patients and could not perform an association analysis of these parameters with the transcriptomic analysis, which would offer some more insights into the dynamic pattern of immune responses during recovery of COVID-19. A focused and well-designed experimental study with rhesus monkey model of SARS-CoV-2 infection would help to clarify these key issues in the future.^29^

In conclusion, we obtained longitudinal whole transcriptome data from patients with different levels of disease severity and allowed us to present a comprehensive view of the dynamics and transcriptional regulation of peripheral blood immune cells during recovery from COVID-19. The rapid attenuation of innate immunity and humoral immunity at the transcription level were important features, and the regulation of poly-functional memory T cell responses was critical for the treatment. The inability to carry out experimental verification is the main limitation, but the mutual verification at the levels of mRNA, miRNA, and lncRNA improved the credibility of the results. These results have direct implications for modulating T cell immunity in the successful treatment of COVID-19 and the development of more effective ways to prevent SARS-CoV-2 infection.

## Materials and methods

### Patient cohort and sample collection

A total of 18 patients with COVID-19 were admitted at the Yunnan Infectious Disease Hospital, Kunming, China, and were enrolled in the study from January 17 to April 9, 2020. Peripheral venous blood samples from patients were obtained at the three clinical stages (treatment, convalescence and rehabilitation). PBMC samples were isolated from the fresh peripheral blood by Ficoll-Paque (GE Healthcare) density gradient centrifugation, and were stored at −80 °C until the use for total RNA extraction. The detailed clinical features of all patients and the detailed sampling time are shown in Fig. 1 and Supplementary Table 1. All samples are processed in a BSL-2 laboratory qualified for SARS-CoV-2 testing, and in accordance with the laboratory biosafety guide for the novel coronavirus (2nd edition) issued by the National Health Commission of China.

### RNA extraction and library preparation

Total RNA was extracted by using the RNAeasy kit (TianGen, Beijing, China) according to the manufacturer’s instructions. The purity, concentration, and integrity of total RNA were checked using the NanoPhotometer spectrophotometer (IMPLEN, CA, USA), the Qubit RNA Assay Kit in Qubit 2.0 Fluorometer (Life Technologies, CA, USA), and the RNA Nano 6000 Assay Kit of the Bioanalyzer 2100 System (Agilent Technologies, CA, USA), respectively. Besides, RNA degradation and contamination were monitored on 1% agarose gels. A total amount of 1 μg total RNA per sample was used to prepare for the rRNA-depleted cDNA library. Ribosomal RNA was removed by Epicentre Ribo-zeroTM rRNA Removal Kit (Epicentre, USA), and rRNA free residue was cleaned up by ethanol precipitation. Subsequently, sequencing libraries were generated using the rRNA-depleted RNA by NEBNext UltraTM Directional RNA Library Prep Kit for Illumina (NEB, USA) and sequenced on an Illumina HiSeq 4000 platform to generate 150 bp paired-end reads. For a small RNA library, a total amount of 2 μg total RNA per sample was used as the input material. Sequencing libraries were generated using NEBNext Multiplex Small RNA Library Prep Set for Illumina (NEB, USA) following the manufacturer’s recommendations. Small RNA libraries were sequenced on an Illumina Hiseq 2500 platform and 50 bp single-end reads were generated. All the data have been deposited in the Gene Expression Omnibus database under the accession number GSE157859.

### RNA sequencing (RNA-seq) data processing

For mRNA and lncRNA, raw RNA-seq reads generated from rRNA-depleted libraries were trimmed to remove sequencing adapters and low-quality reads. Quality of data were checked by FastQC v0.11.9. Adapters were removed by Trimmomatic v0.39.^54^ The clean reads were then aligned to the primary assembly of the human reference genome, GRCh38, using STAR v2.7.3a.^55^ StringTie v2.1.2 was used to assemble transcripts in a reference-guided manner for each sample.^56^ The reference (GENCODE v33) and assembled transcripts for all samples were merged by StringTie.^56^ Novel transcripts were obtained by comparing the merged transcripts with the reference transcripts by cuffcompare (code==‘x’ | Code==‘u’ | code==‘i’).^57^ Novel lncRNAs were identified through the following filters: (1) transcript length >200 bp and >=2 exons; (2) lack of coding potential predicted by both CPC2^58^ and CPAT;^59^ (3) no overlap with known lncRNAs from the RefLnc^60^ or LNCipedia databases.^61^ Novel lncRNAs, known lncRNAs from RefLnc and LNCipedia,^61^ and reference transcripts (GENCODE v33) were merged and used for transcript quantification by kallisto v0.46.1.^62^ For small RNA-seq analysis, known and novel miRNAs were obtained and quantified by miRdeep2^63^ using human and chimpanzee miRNA from miRBase v22.1^64^ as the references.

### Differentially express RNA and target genes for non-coding RNA

DESeq2^65^ was used to identify differentially expressed mRNAs (DEGs), lncRNAs (DElncRNAs) or miRNAs (DEmiRNAs) between patients at different clinical stages or with different clinical types (Supplementary Data 1, 2). *P*-value of differential expression was adjusted by the Benjamini–Hochberg’s (BH) method (FDR). Because some samples with poor library quality were excluded, DEGs and DElncRNAs were calculated based on 39 samples, while DEmiRNAs was calculated based on all samples. The target genes of lncRNAs was predicted by their nearest and co-expressed (Pearson’s correlation *P* < 0.05) protein-coding genes. Target genes of miRNA were obtained by merging the results from two publicly available databases miRDB^66^ and miRWalk 2.0.^67^ Genes predicted by both of the two databases and negatively co-expressed (Pearson’s correlation *P* < 0.05 and *r* < 0) with the miRNA were defined as target genes of the miRNA. The functional annotation and analysis of DEmiRNA and DElncRNA in this study are for their target genes, therefore non-coding RNAs without target genes were excluded from functional analysis.

### Gene set enrichment analysis (GSEA)

The values of log_2_ fold change relative to the treatment stage of DEG and target genes for DEmiRNA and DElncRNA at the convalescence stage and the rehabilitation stage are subjected to linear regression analysis. Then through the GSEA algorithm of DOSE,^68^ the slope values of log_2_ fold change of these genes are used to calculate the significantly enriched biological process GO gene sets (FDR < 0.05) and the corresponding enrichment scores. These GSEA results were further classified and summarized by REVIGO,^69^ and converted into two-dimensional lattice data based on GO semantic space. Finally, clustering algorithms are used to analyze the similarities and differences of the signal pathways affected by different types of transcriptomes (mRNA, miRNA, and lncRNA) or different clinical types of samples (Mild, moderate, and severe).

### Functional analyses

Since there are multiple comparisons of samples at three clinical stages in our study, there can be as many as 8 differential expression patterns of the differentially expressed transcriptome during COVID-19 recovery. DEGs and target genes for DEmiRNAs or DElncRNAs can be further divided into interferon-stimulated genes (ISG), transcription factor genes and phosphorylation regulatory genes according to their functions. These differentially expressed RNAs with different expression patterns and functions are respectively analyzed and visualized for GO enrichment by clusterProfiler.^70^ In addition, clusterProfiler and pathview^71^ can further construct KEGG enrichment network and functional network of these genes, and visualize the relationship between genes and between genes and signal pathways. All differentially expressed RNAs are constructed to a protein–protein interaction network (PPI) by STRING V11.^72^ According to the network analysis, we select 65 highly differentially expressed (Log_2_ fold change >1) genes with top connectivity and 19 related non-coding RNAs to reconstruct a core PPI network.

### Weighted correlation network analysis (WGCNA) network construction

The mRNA, lncRNA, and miRNA signed WGCNA co-expression network was constructed on the basis of the top 8000 most variably expressed mRNAs/lncRNAs and top 600 most variably expressed miRNAs in all patients with COVID-19. The correlation matrix was obtained by calculating the Pearson’s correlations between all gene pairs across all subjects in the dataset, and then were converted into an adjacency matrix using a power function (power β = 14). For module detection, the adjacency matrix was further transformed into a topological overlap matrix (TOM), and hierarchical clustering was used to group genes based on the dissimilarity matrix (1-TOM), followed by a dynamic cut-tree algorithm to dynamically cut clustering dendrogram branches into gene modules (Supplementary Fig. 8). A height cutoff of 0.1 was used to merge modules whose expression profiles are highly similar (Supplementary Data 3). Correlations between module eigengenes and traits of patients with COVID-19 were computed by Pearson’s correlation.^73,74^ Network was visualized by using the Cytoscape 3.8 software.^75^

## Supplementary information

Supplementary materials

Dataset 1

Dataset 2

Dataset 3

## Data Availability

All the RNA sequencing data have been deposited in the Gene Expression Omnibus database under the accession number GSE157859.
