# Supplementary material for: Longitudinal transcriptome analyses show robust T cell immunity during recovery from COVID-19
Source: Signal Transduct Target Ther. 2020 Dec 24;5:294. doi: 10.1038/s41392-020-00457-4 (PMC7758413; doi:10.1038/s41392-020-00457-4)
Supplement: Supplementary file 1 — Supplementary materials [file 41392_2020_457_MOESM1_ESM.pdf]

Supplementary Materials for

Longitudinal transcriptome analyses show robust T cell immunity during recovery from COVID-19

Hong-Yi Zheng, Min Xu, Cui-Xian Yang, Ren-Rong Tian, Mi Zhang, Jian-Jian Li, Xi-Cheng Wang, Zhao-Li Ding, Gui-Mei Li, Xiao-Lu Li, Yu-Qi He, Xing-Qi Dong, Yong-Gang Yao, Yong-Tang Zheng

Correspondence to: zhengyt@mail.kiz.ac.cn

This PDF file includes:

Figures. S1 to S8
Tables S1
Captions for Data S1 to S3

Other Supplementary Materials for this manuscript include the following:

Data S1 to S3 [S1:DESeq2 results to identify differentially expressed genes and lncRNAs; S2: DESeq2 results to identify differentially expressed miRNAs; S3: WGCNA results]

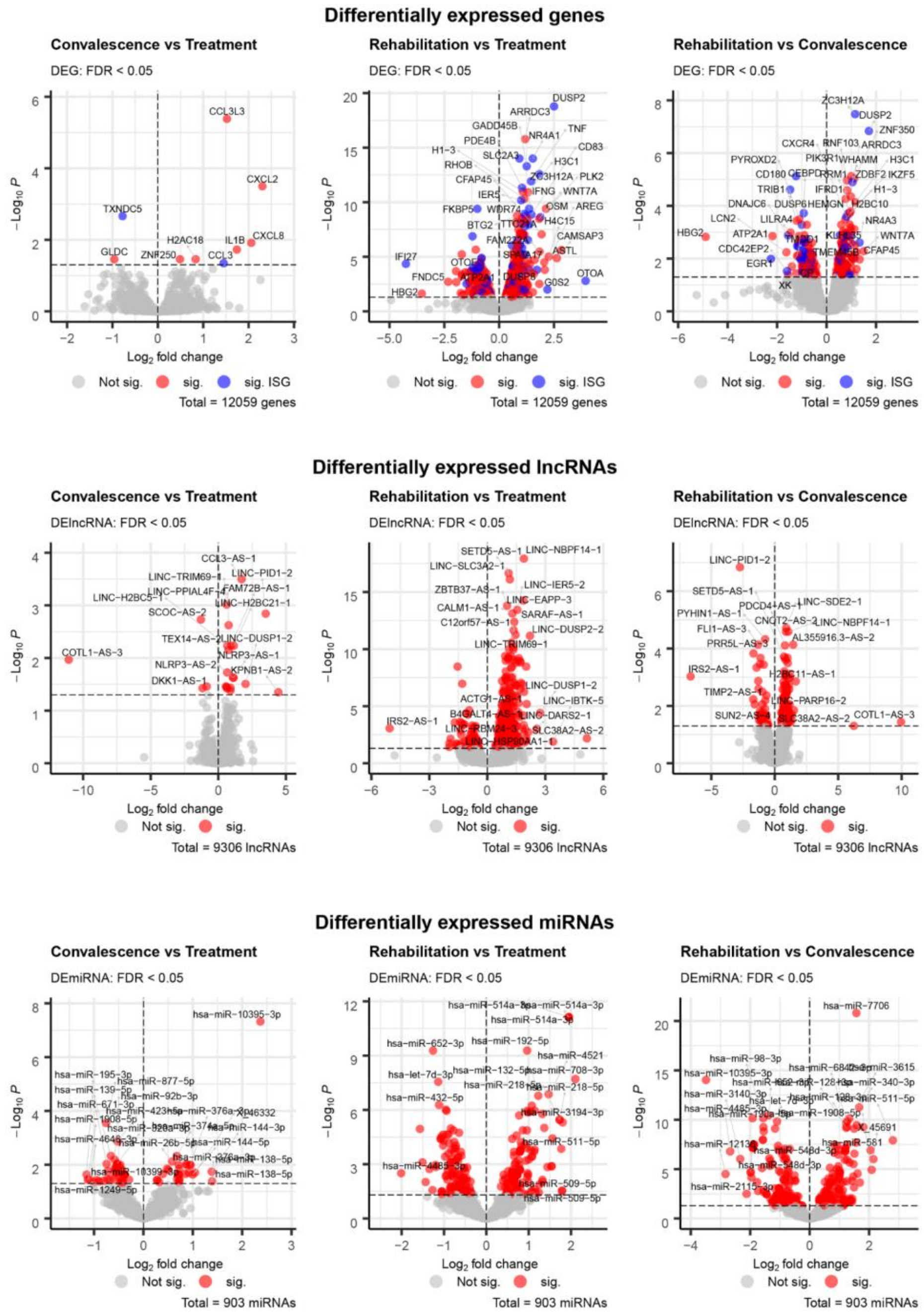

Figure. S1.

The volcano plots display differentially expressed genes (DEG), miRNAs (DEmiRNA) and lncRNAs (DElncRNA).

Each plot shows the log2 fold change and-log10 FDR of each gene, miRNA or lncRNA calculated after pairwise comparison of samples in the treatment, convalescence and rehabilitation stage. For differential expression, FDR < 0.05 is selected.

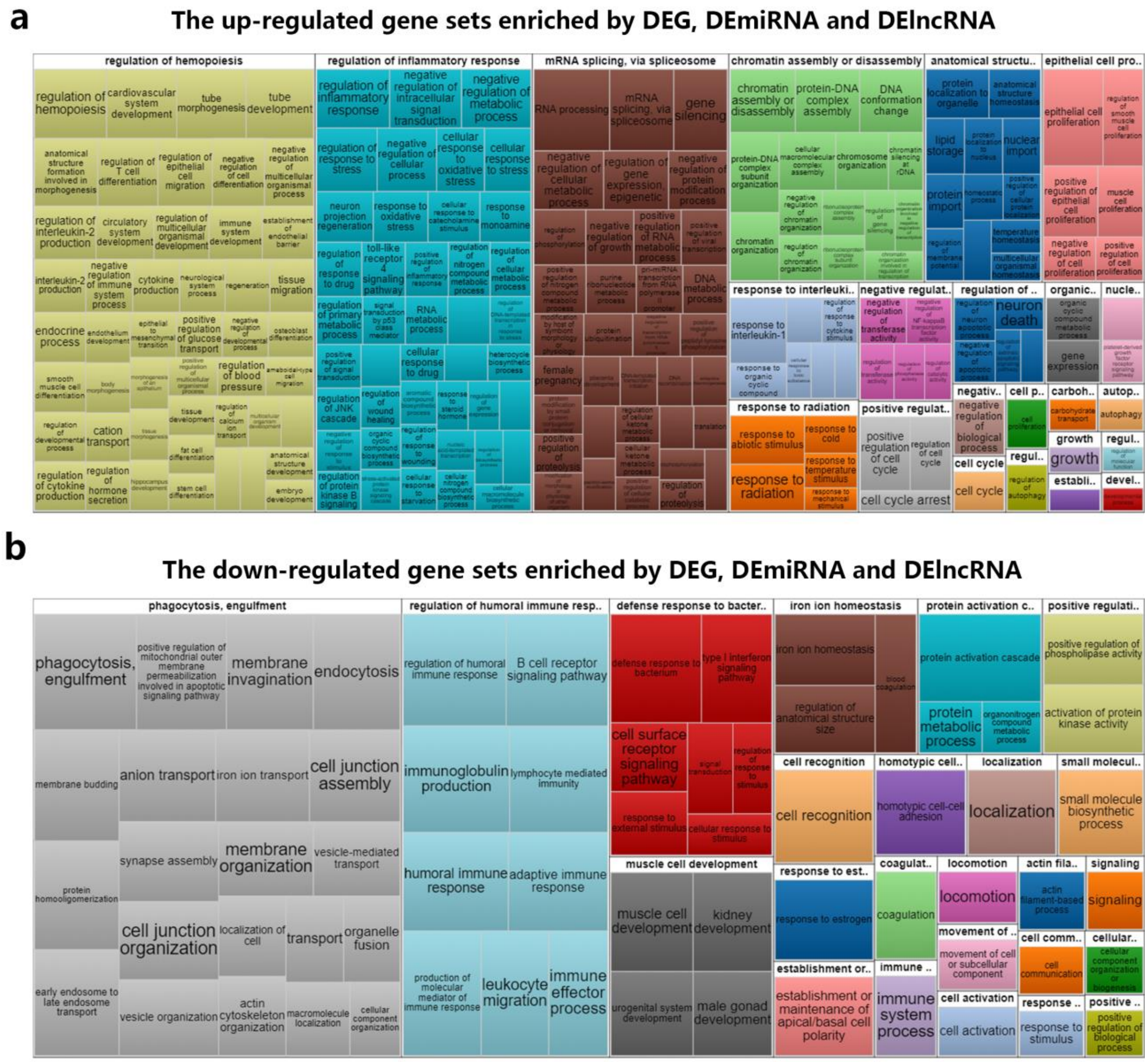

Figure. S2.

The treemaps show the gene ontology (GO) semantic classification results of the gene sets enriched by DEG, DEmiRNA and DElncRNA.

a, b Differentially expressed genes and non-coding RNAs were subjected to gene set enrichment analysis (GSEA) to obtain the GO terms and enrichment scores of the up-regulated (**a**) or down-regulated (**b**) gene sets. Then these enrichment results are analyzed by REViGO package, and similar GO terms are merged and summarized. The color represents GO items in the same category, and the size reflects the relative enrichment score of the GO term.

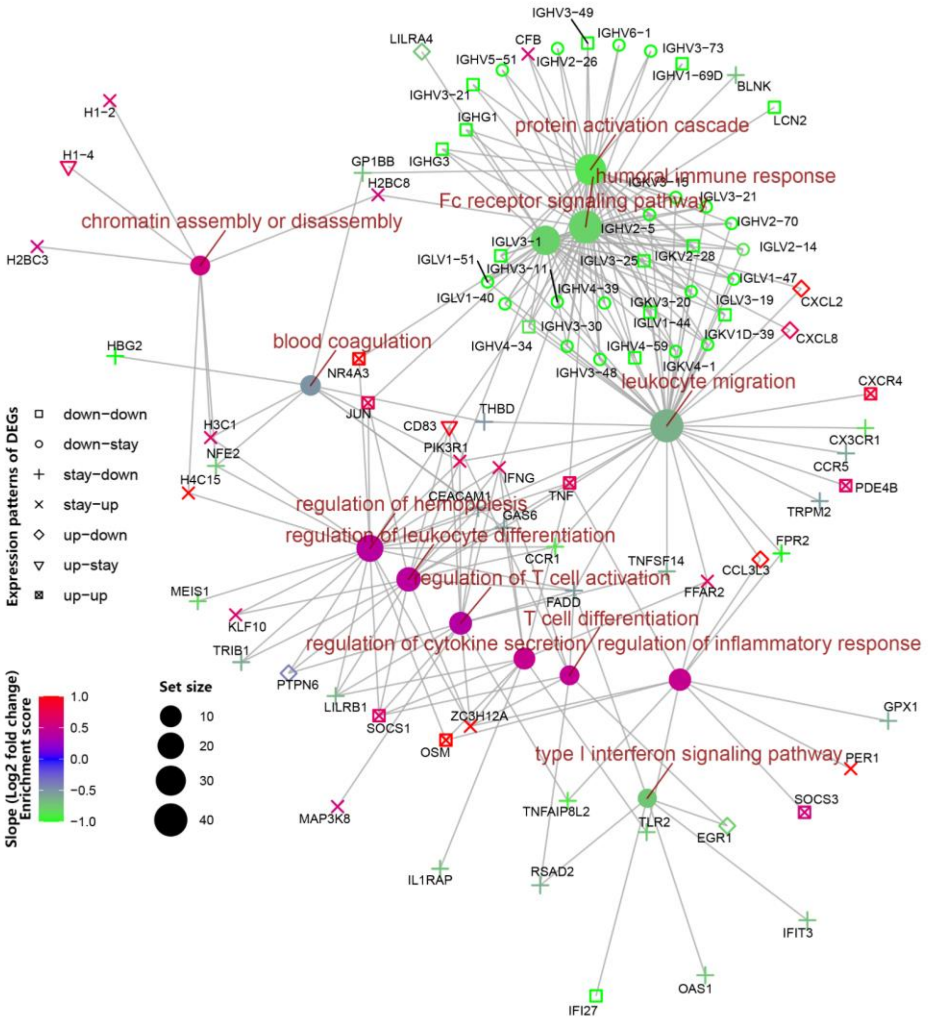

Figure. S3.

Representative GO enrichment networks of DEGs.

We selected the DEGs with high connection numbers and the absolute value of Log2 fold change greater than 1 in the results of GSEA to construct GO enrichment networks. The color of the gene set reflects its enrichment score, and the size reflects the number of its enriched genes. The color of the gene reflects the slope of the linear regression equation of Log2 fold change, and the shape reflects its expression pattern during recovery from COVID-19. By comparing the P value and TPM/CPM at three different clinical stages, DEGs, DEmiRNAs or DElncRNAs can be further clustered into 8 expression patterns (down-down, down-stay, down-up, stay-down, stay-up, up-down, up-stay, up-up). Each pattern reflects the characteristics of its RNAs being up-regulated (up), down-regulated (down) or stayed for no change (stay) at the convalescence and rehabilitation stages relative to the treatment stage.

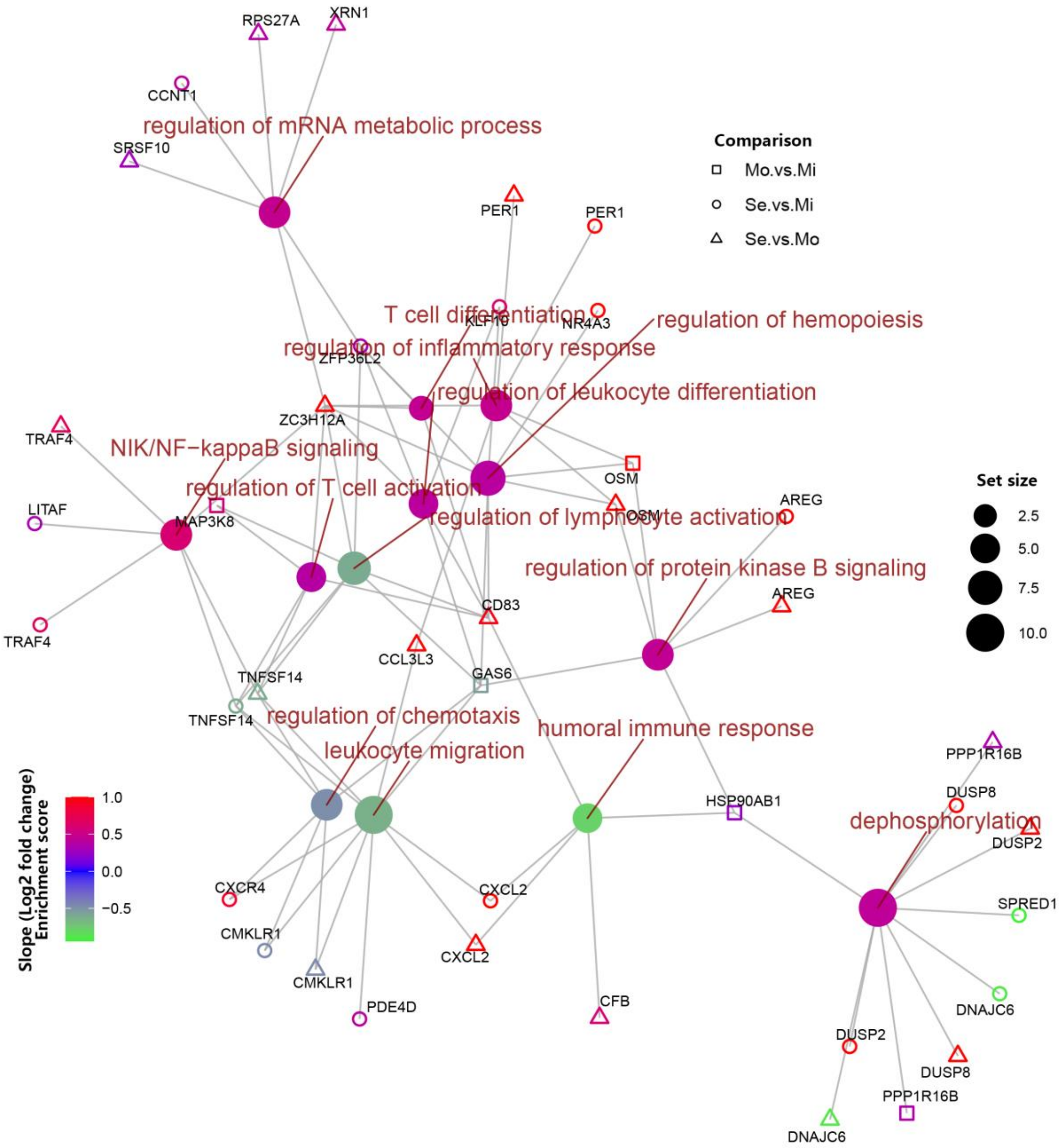

Figure. S4.

Representative GO enrichment networks of clinical type related DEGs.

We selected the DEGs between three clinical types (Mi, mild; Mo, moderate; Se, severe) to construct GO enrichment networks. The color of the gene set reflects its enrichment score, and the size reflects the number of its enriched genes. The color of the gene reflects the slope of the linear regression equation of Log2 fold change during recovery from COVID-19, and the shape reflects the comparison between which two groups it came from.

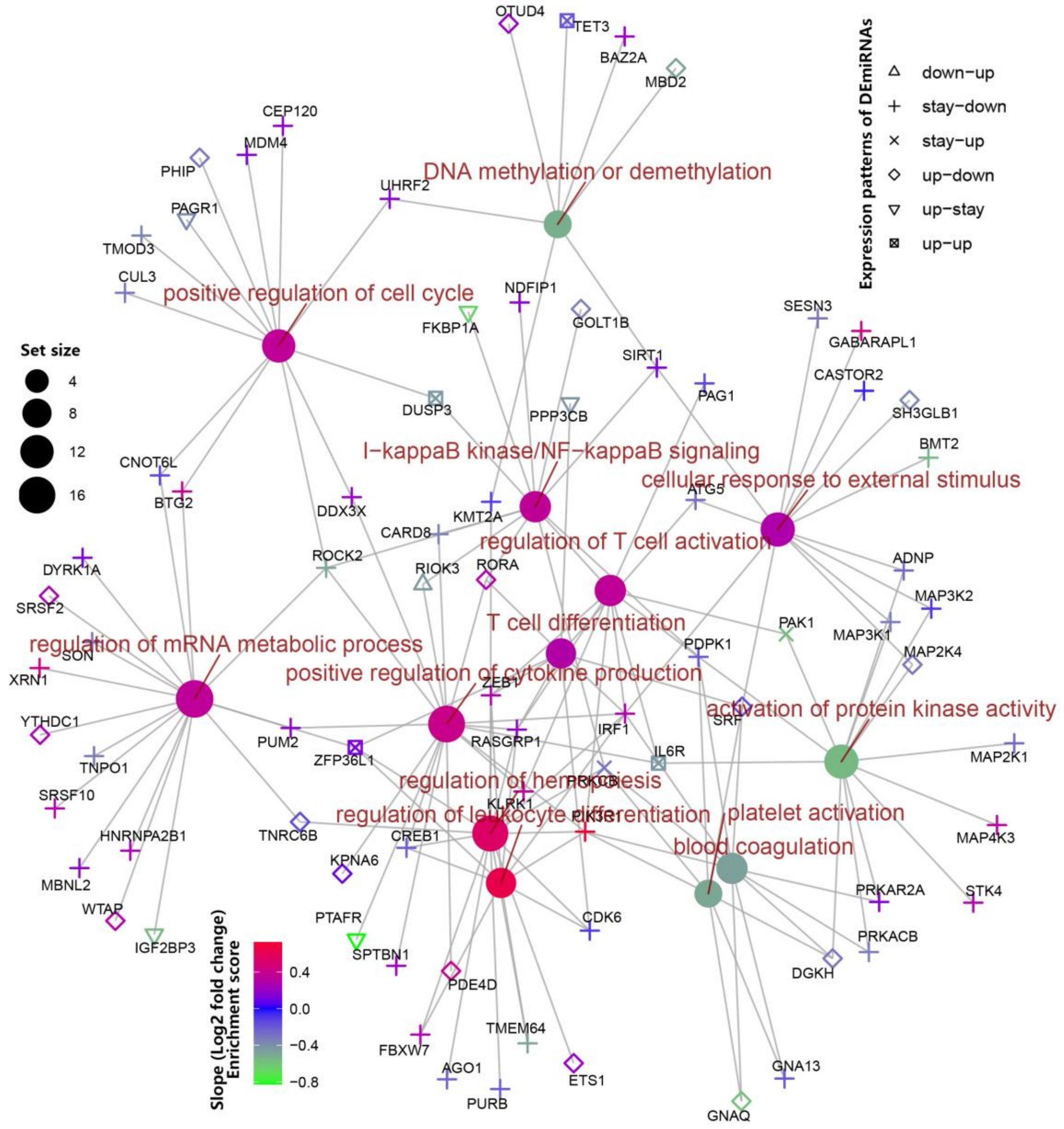

Figure. S5.

Representative GO enrichment networks of DEmiRNA target genes.

We selected the DEmiRNA target genes with high connection numbers and the absolute value of Log2 fold change greater than 1 in the results of GSEA to construct GO enrichment networks. The color of the gene set reflects its enrichment score, and the size reflects the number of its enriched genes. The color of the gene reflects the slope of the linear regression equation of Log2 fold change, and the shape reflects the expression pattern of its related DEmiRNA during recovery from COVID-19.

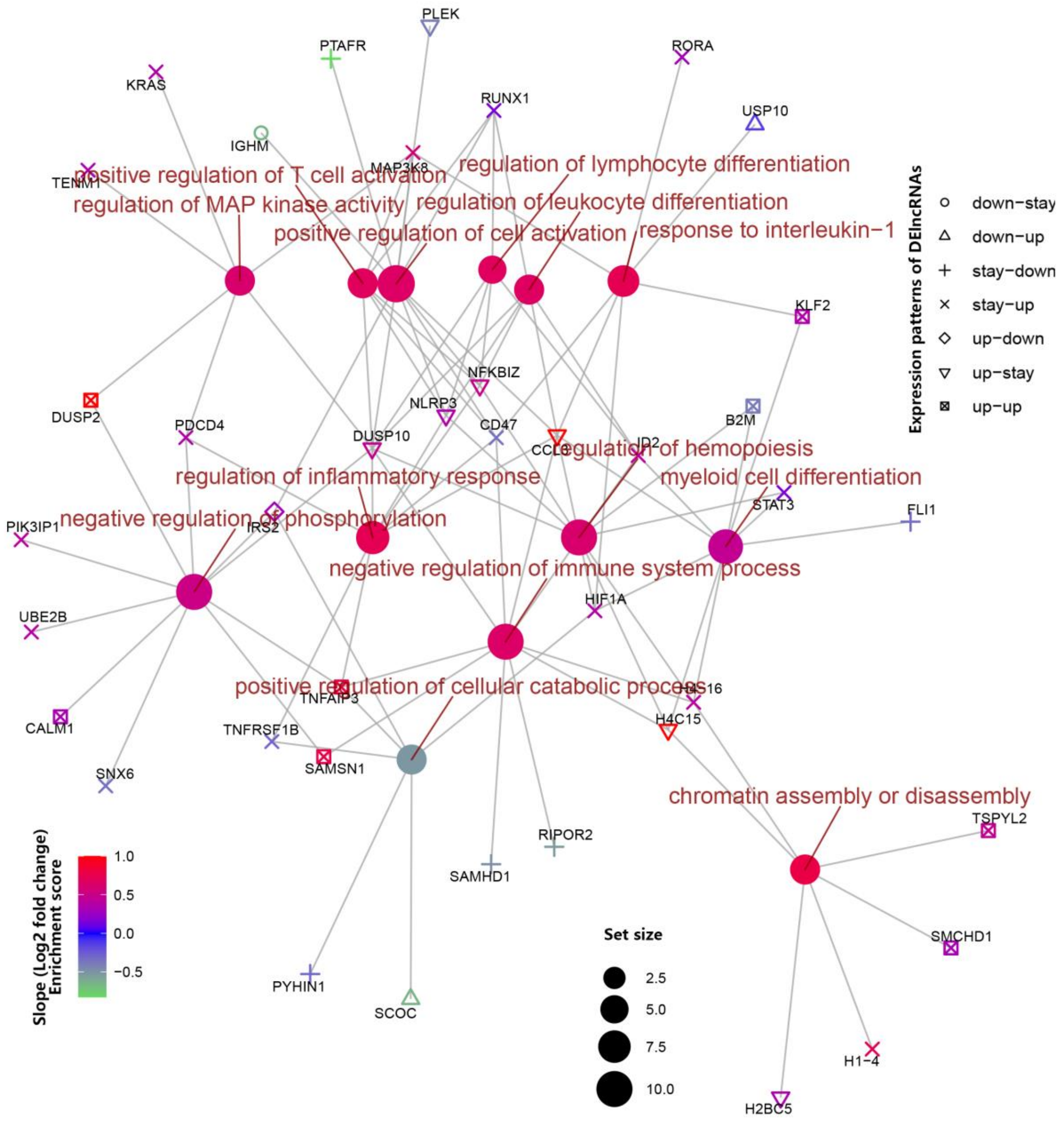

Figure. S6.

Representative GO enrichment networks of DElncRNA target genes.

We selected the DElncRNA target genes with high connection numbers and the absolute value of Log2 fold change greater than 1 in the results of GSEA to construct GO enrichment networks. The color of the gene set reflects its enrichment score, and the size reflects the number of its enriched genes. The color of the gene reflects the slope of the linear regression equation of Log2 fold change, and the shape reflects the expression pattern of its related DElncRNA during recovery from COVID-19.

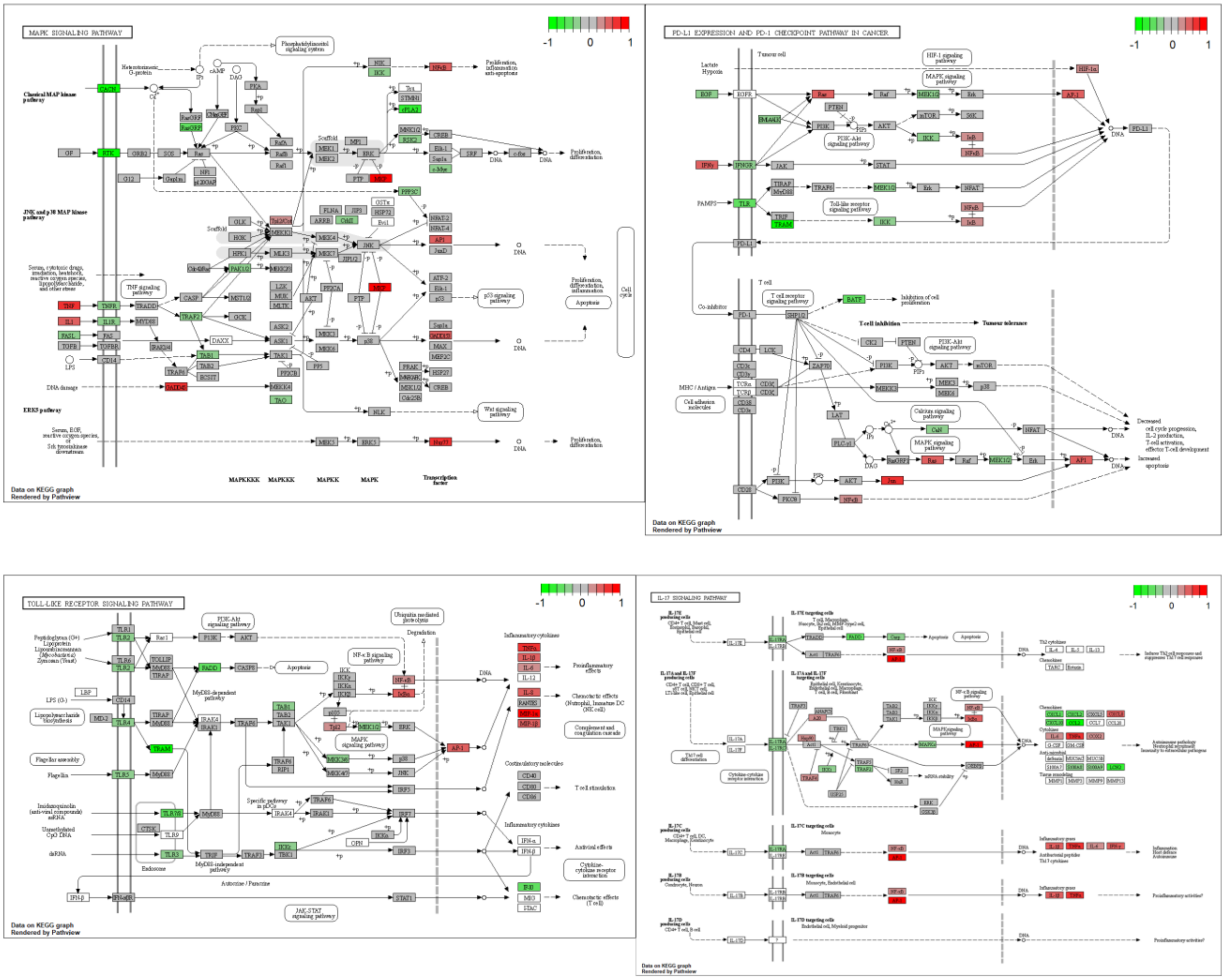

Figure. S7.

KEGG graphs show the interaction of differentially expressed genes in different signaling pathways.

The color indicates linear regression slope of Log2 fold change of a gene.

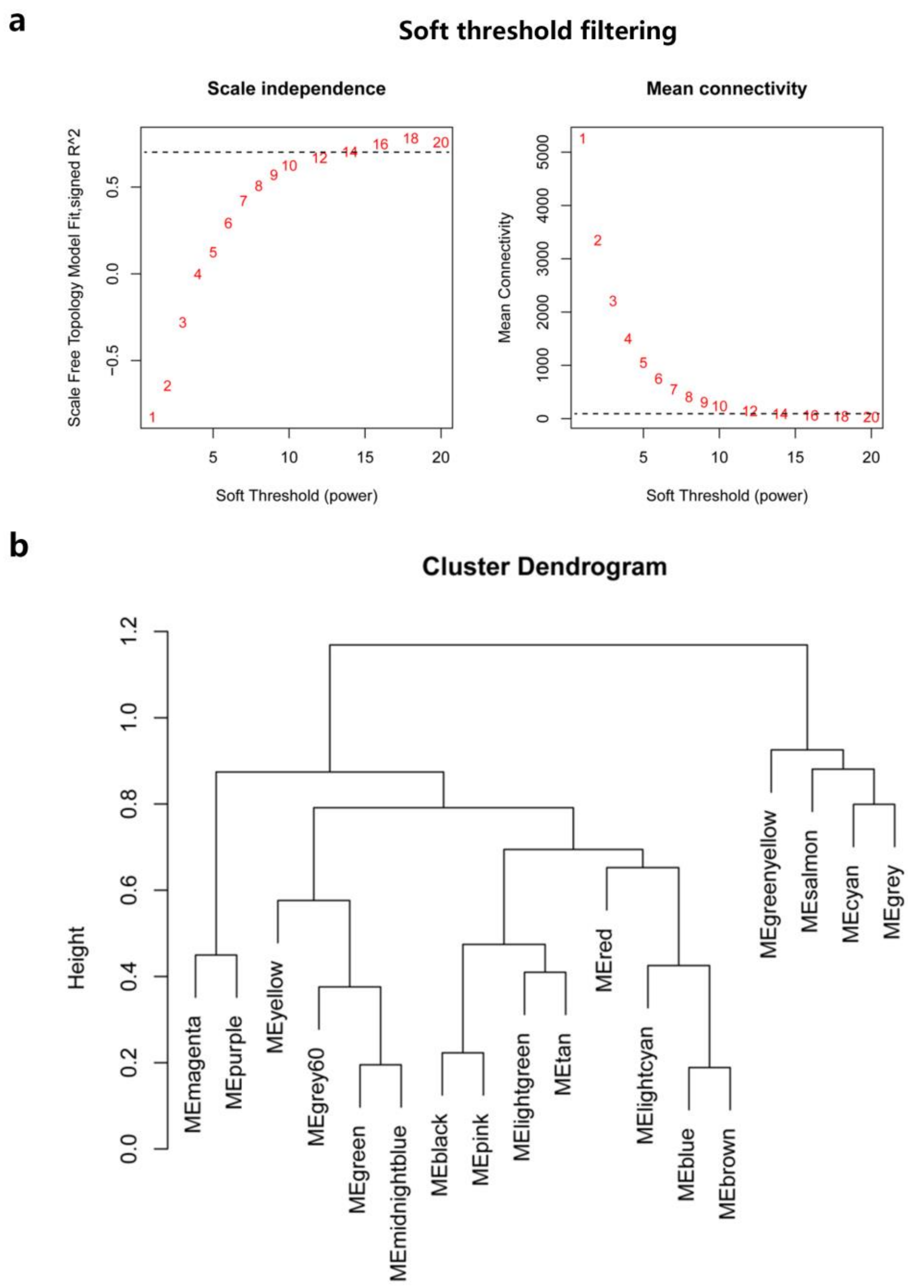

Figure. S8.

Soft threshold filtering and module clustering in Weighted Gene Co-Expression Network Analysis (WGCNA).

a The optimal soft threshold was determined based on the criterion of approximate scale-free topology by using the pickSoftThreshold function. **b** According to the soft threshold, WGCNA produced 18 co-expression modules. Hierarchical clustering shows the relationship between these modules.

Table S1.

Demographic and clinical data of patients with COVID-19.

Name	ID	Age (years)	Gender	Main comorbidities	Clinical type	Therapy method	blood samples available at clinical stages		
							**T**	**C**	**R**
**LY**	1	28	M	-	mild	Lopinavir/Ritonavir + IFNα-2b	√	√	-
**QY**	2	36	F	-	mild	Lopinavir/Ritonavir + IFNα-2b	√	√	√
**HY**	3	40	M	-	mild	IFNα-2b	√	√	√
**YZY**	4	3.5	M	-	mild	IFNα-2b	√	√	√
**WSS**	5	22	F	-	mild	IFNα-2b	√	√	-
**HWZ**	6	10.8	M	-	mild	IFNα-2b	√	√	-
**FHW**	7	58	M	-	moderate	Lopinavir/Ritonavir + IFNα-2b	√	√	√
**YGT**	8	56	F	hypertension	moderate	Lopinavir/Ritonavir + IFNα-2b	√	√	√
**YL**	9	39	F	-	moderate	Lopinavir/Ritonavir + IFNα-2b	√	√	√
**WHX**	10	49	F	-	moderate	Lopinavir/Ritonavir + IFNα-2b	√	√	√
**XF**	11	32	F	-	moderate	Lopinavir/Ritonavir + IFNα-2b	√	√	√
**LSH**	12	52	M	type II diabetes, HCV	moderate	Abidor hydrochloride + Lopinavir/Ritonavir	√	√	-
**LZL**	13	58	M	-	moderate	Lopinavir/Ritonavir + IFNα-2b	√	√	-
**GCY**	14	69	F	hypertension	severe	ribavirin + Lopinavir/Ritonavir + IFNα-2b	√	√	√
**WWL**	15	58	M	type II diabetes	severe	Lopinavir/Ritonavir + IFNα-2b	√	√	√
**ZZB**	16	50	M	hypertension	severe	IFNα-2b	√	√	-
**YBL**	17	64	M	hypertension,after myocardial infarction operation	severe	Lopinavir/Ritonavir + IFNα-2b	-	√	√
**WJH**	18	49	M	hypertension	severe	Abidor hydrochloride + Lopinavir/Ritonavir	√	√	√
	**summary**	**43.0±18.2**	**61.1% male<br>38.9% female**	**38.9% comorbidities**	**33.3% mild<br>38.9% moderate<br>27.8 severe**	**88.9% IFNα-2b**	**17**	**18**	**12**

M, male. F, Female. T, treatment stage. C, convalescence stage. R, rehabilitation stage. "√" refers to the available of the sample, otherwise marked with "-".

Data S1. (separate file)

DESeq2 results to identify differentially expressed genes and lncRNAs.xlsx

The Excel table shows the comparison between the different clinical stages of each mRNA and lncRNA by DESeq2 package, including log2FoldChange, p value and FDR

Data S2. (separate file)

DESeq2 results to identify differentially expressed miRNAs. xlsx

The Excel table shows the comparison between the different clinical stages of each miRNA, by DESeq2 package, including log2FoldChange, p value and FDR

Data S3. (separate file)

WGCNA results.xlsx

The Excel table shows the co-expression modules and connectivity of each gene by Weighted correlation network analysis (WGCNA).
